# scEnhancer: a single-cell enhancer resource with annotation across hundreds of tissue/cell types in three species

**DOI:** 10.1093/nar/gkab1032

**Published:** 2021-11-11

**Authors:** Tianshun Gao, Zilong Zheng, Yihang Pan, Chengming Zhu, Fuxin Wei, Jinqiu Yuan, Rui Sun, Shuo Fang, Nan Wang, Yang Zhou, Jiang Qian

**Affiliations:** Big Data Center, The Seventh Affiliated Hospital of Sun Yat-sen University, Shenzhen 518107, P.R. China; Scientific Research Center, The Seventh Affiliated Hospital of Sun Yat-sen University, Shenzhen 518107, P.R. China; Big Data Center, The Seventh Affiliated Hospital of Sun Yat-sen University, Shenzhen 518107, P.R. China; Big Data Center, The Seventh Affiliated Hospital of Sun Yat-sen University, Shenzhen 518107, P.R. China; Scientific Research Center, The Seventh Affiliated Hospital of Sun Yat-sen University, Shenzhen 518107, P.R. China; Scientific Research Center, The Seventh Affiliated Hospital of Sun Yat-sen University, Shenzhen 518107, P.R. China; Department of Orthopaedics, The Seventh Affiliated Hospital of Sun Yat-sen University, Shenzhen 518107, P.R. China; Big Data Center, The Seventh Affiliated Hospital of Sun Yat-sen University, Shenzhen 518107, P.R. China; Scientific Research Center, The Seventh Affiliated Hospital of Sun Yat-sen University, Shenzhen 518107, P.R. China; Big Data Center, The Seventh Affiliated Hospital of Sun Yat-sen University, Shenzhen 518107, P.R. China; Scientific Research Center, The Seventh Affiliated Hospital of Sun Yat-sen University, Shenzhen 518107, P.R. China; Big Data Center, The Seventh Affiliated Hospital of Sun Yat-sen University, Shenzhen 518107, P.R. China; Department of Oncology, The Seventh Affiliated Hospital of Sun Yat-sen University, Shenzhen 518107, P.R. China; Scientific Research Center, The Seventh Affiliated Hospital of Sun Yat-sen University, Shenzhen 518107, P.R. China; Big Data Center, The Seventh Affiliated Hospital of Sun Yat-sen University, Shenzhen 518107, P.R. China; The Wilmer Eye Institute, Johns Hopkins School of Medicine, Baltimore, MD 21231, USA; The Sidney Kimmel Comprehensive Cancer Center, Johns Hopkins School of Medicine, Baltimore, MD 21205, USA

## Abstract

Previous studies on enhancers and their target genes were largely based on bulk samples that represent ‘average’ regulatory activities from a large population of millions of cells, masking the heterogeneity and important effects from the sub-populations. In recent years, single-cell sequencing technology has enabled the profiling of open chromatin accessibility at the single-cell level (scATAC-seq), which can be used to annotate the enhancers and promoters in specific cell types. A comprehensive resource is highly desirable for exploring how the enhancers regulate the target genes at the single-cell level. Hence, we designed a single-cell database scEnhancer (http://enhanceratlas.net/scenhancer/), covering 14 527 776 enhancers and 63 658 600 enhancer-gene interactions from 1 196 906 single cells across 775 tissue/cell types in three species. An unsupervised learning method was employed to sort and combine tens or hundreds of single cells in each tissue/cell type to obtain the consensus enhancers. In addition, we utilized a cis-regulatory network algorithm to identify the enhancer-gene connections. Finally, we provided a user-friendly platform with seven useful modules to search, visualize, and browse the enhancers/genes. This database will facilitate the research community towards a functional analysis of enhancers at the single-cell level.

## INTRODUCTION

The DNA cis-regulatory elements, such as distal enhancers and promoters, determine the transcriptional regulation of tissue/cell type-specific gene expression in development, embryogenesis, immunity, homeostasis and diseases (1–5). To date, the annotation for enhancers or super-enhancers increased rapidly through many large-scale resources, including EnhancerAtlas, SEA, Endb, CancerEnD, SEdb, HACER, RAEdb, HEDD, DiseaseEnhancer, GeneHancer, DENdb, dbSUPER and VISTA (6–19). These resources annotate enhancers from bulk datasets and measured only average enhancer activities in large populations of cells, masking the heterogeneity and key effects among and within the sub-populations (e.g. sub-cell types) containing small numbers of cells (3,20–22) (Supplementary Figure S1). Since enhancers are tissue/cell type-specific, enhancer identification based on the single-cell level can better reveal the cellular specificity to determine the differences of gene expression on phenotypes across tissue/cell types (23,24). Therefore, single-cell resources are ideal for exploring how the enhancers regulate the target genes at ultra-high resolution in an accurate cell type-specific manner.

Assay of Transposase-Accessible Chromatin using sequencing (ATAC-seq) is a powerful tool for epigenomic profiling of cell type-specific chromatin accessibility (25). It was reported that at least 50% and around 25% of the bulk ATAC-seq peaks fell into the enhancer and promoter regions, respectively (26). Thus, the peaks called from ATAC-seq mainly represent cis-regulatory elements, including enhancers and promoters, and can be used to annotate tissue/cell type-specific enhancers or promoters (22,27,28). At the single-cell level, scATAC-seq studies have been widely applied to identify cell type-specific enhancers or enhancer–ṇpromoter interactions (1,3,4,29–34). Especially, accessible sites in the human genome identified by scATAC-seq displayed a high overlap with 75% of experimentally validated active enhancers in VISTA (3,9). Using the Graphical LASSO, a single-cell cis-regulatory network algorithm, Cicero, was developed (33) and enabled identification of genome-wide enhancer–ṇpromoter connections on a large scale (1,3,4,29–32,34). For example, Domcke *et al* utilized Cicero to identify 6.3 million unique pairs of cis-regulatory elements in 54 human cell types (3). These indicate that scATAC-seq may be an ideal single-cell sequencing technique for annotating enhancers and enhancer–ṇpromoter interactions.

Here, we constructed a single-cell enhancer database, scEnhancer, based on an improved unsupervised learning approach previously developed in our bulk enhancer database, EnhancerAtlas 2.0 (6). This method was used to integrate many genomic datasets to derive a consensus annotation of enhancers. It displayed several characteristics: (i) it was based on a well-designed score voting strategy for ranking and combining a large set of unlabelled data (7,35); (ii) we replaced the Pearson correlation with the Jaccard index, which was appropriate for the binary nature of scATAC-seq data, for computing similarity among all single-cell datasets (36); (iii) in contrast to the only 12 independent high-throughput datasets used in EnhancerAtlas 2.0, the new method could process tens or hundreds of single-cell datasets with different qualities as measured by the average number of fragments per cell; (iv) the Cicero results were used as a filtering condition for identification of the final single-cell enhancers (33). We also leveraged Cicero to generate enhancer–ṇpromoter connections of high quality (1,3,4,29–32,34). In some aspects, as a comprehensive single-cell enhancer resource, scEnhancer possesses tremendous advantages: (i) it has profiled 1 196 906 single cells and annotated a total of 14 527 776 enhancers in 775 tissue/cell types across three species; (ii) a suitable combination of an improved unsupervised learning method and a cis-network algorithm was applied to identify the enhancers and enhancer-gene interactions and (iii) a user-friendly platform with seven functional modules and the browser options were designed for searching, visualizing, drawing, and browsing enhancer or enhancer–ṇpromoter profiles. These will facilitate the analysis of enhancers at the single-cell level for the research community.

## MATERIALS AND METHODS

### Single-cell data collection and integration

To identify single-cell enhancers, we collected raw or processed (e.g. by cellranger) single-cell datasets with peak and tissue/cell-type annotations from several scATAC-seq resources, including the NCBI GEO datasets (37), Signac analysis with 10X Genomics data (38), DESCARTES (3), LungMap (39), Mouse sci-ATAC-seq Atlas (4), Fly ATAC Atlas (1) and MPAL-Single-Cell-2019 (29). All the samples in human, mouse, and fly were mapped to genome builds GRCh37/hg19, GRCm37/mm9 and BDGP5/dm3, respectively, by liftOver (40).

### Integration of tissue/cell-type specific binary matrix

To obtain the tissue/cell-type specific binary matrix, we first converted the processed or raw scATAC-seq data into a large standard matrix with labelled cell types. In most scATAC-seq projects, the single-cell data are usually presented in many file formats, such as MatrixMarket (4), h5 (38), txt (41), RangedSummarizedExperiment (42), Seurat RDS (3), or even the raw fastq files (43). Here, we used the functions in R language and cellranger-atac 1.2.0 (30) to transform these different formats of single-cell data into large standard matrices for the subsequent extraction of small cell-type specific matrices (Supplementary Table S1). Data in MatrixMarket or h5 formats were transformed into the standard matrix via ‘Matrix::readMM’ and ‘Read10X_h5’ in Signac (38) while the txt datasets could be read as data.frame and then converted into the matrices. For the RangedSummarizedExperiment dataset, we first parsed its R structure to obtain the matrix, peaks, cell barcodes and cell type annotations from its ‘assay’, ‘colnames’, ‘colData’, ‘rowRanges’ slots (42). Analogously, we parsed the dataset in Seurat RDS and extracted the matrix with peak, barcode, and cell type information from the slots ‘GetAssayData’, ‘assays RNA/peaks’ and ‘meta.data’, respectively (3). The raw fastq single-cell datasets could be transformed into a large standard matrix by cellranger-atac (30) that processes peak and cell callings into MatrixMarket or h5 formats (43). Finally, we removed the irregular datasets with fewer than 200 peaks. Signac and cellranger-atac tools can be downloaded and installed from: https://satijalab.org/signac/ and https://support.10xgenomics.com/single-cell-atac/software/downloads/1.2.

After integrating the large standard matrix containing barcodes of all mixed cell types, we extracted cell-type specific single-cell datasets from the matrix based on the cell type annotation information in the metadata. To make the single-cell datasets comparable, we binarized them to normalize each dataset. In each dataset, the peak signal was set to ‘1’ (‘open’) for at least one read and ‘0’ (‘closed’) otherwise in the absence of reads (36). Thus, all the single-cell datasets for one tissue/cell type were merged and consolidated into a binary cell-type matrix. In this cell-type matrix, single cells (i.e. columns) with less than 200 peaks (i.e. rows) were removed, as well as the peaks without any signal in all single cells or in uncommon chromosomes (e.g. chr1_random). We also removed the cell types with <50 single cells. Genomic coordinates of peaks with other genome builds in human and mouse were converted by liftOver to GRCh37/hg19 and GRCm37/mm9, respectively (40).

### Generation of consensus single-cell enhancers

We designed an improved unsupervised method to identify the bulk consensus enhancers from 12 types of independent datasets in EnhancerAtlas 2.0 (6). Here, we modified the method to determine the weights of hundreds of single-cells and combine them to generate consensus single-cell enhancers. On average, there were hundreds of single cells per cell type in our integrated data (Table 1). For these datasets we hypothesized that one dataset was high-quality if it was highly correlated with the other datasets and low-quality otherwise (7). Since single-cell datasets are binary, to weigh more on the ‘open’ peaks across the whole genome rather than the ‘close’ peaks, we applied the Jaccard coefficient that had been used for scATAC-seq clustering analysis (36,44), to measure the correlation between any two single cells (e.g. ${c_i}$and ${c_j}$) based on the overlapping degree in open chromatin regions:

$$\begin{equation*}{\rm{ }}{J_{{C_i}{C_j}}} = \frac{{\left| {{C_i} \cap {C_j}} \right|}}{{\left| {{C_i} \cup {C_j}} \right|}}{\rm{ }} = \frac{{\left| {{C_i} \cap {C_j}} \right|}}{{\left| {{C_i}} \right| + |{C_j}\left| - \right|{C_i} \cap {C_j}|}} \end{equation*}$$

where $| {{{{C}}_{i}}} |$, $|{{{C}}_{{j}}}|$ and $| {{{{C}}_{{i}}} \cap {{{C}}_{{j}}}} |$ represent the number of ‘open’ peaks in ${{{C}}_{i}}$, ${{C}_{j}}$ and their overlap, respectively. For a cell type with $n$ single-cell datasets, a Jaccard similarity matrix for all combined datasets was integrated as:

$$\begin{equation*}\left[ {\begin{array}{@{}*{3}{c}@{}} {\begin{array}{@{}*{3}{c}@{}} {{J_{{C_1}{C_1}}}}& \cdots &{{J_{{C_1}{C_i}}}}\\ \vdots &\ &{\ \vdots } \end{array}}&{\begin{array}{@{}*{2}{c}@{}} \cdots &{{J_{{C_1}{C_n}}}}\\ \ &{\ \vdots \ } \end{array}}\\ {\begin{array}{@{}*{3}{c}@{}} {{J_{{C_i}{C_1}}}}& \cdots &{{J_{{C_i}{C_i}}}}\\ {\ \vdots }&\ & \vdots \\ {{J_{{C_n}{C_1}}}}& \cdots &{{J_{{C_n}{C_i}}}} \end{array}}&{\begin{array}{@{}*{2}{c}@{}} \cdots &{{J_{{C_i}{C_n}}}}\\ \ & \vdots \\ \cdots &{{J_{{C_n}{C_n}}}} \end{array}} \end{array}} \right]\end{equation*}$$

**Table 1.** The numbers of tissue/cell types, single-cell enhancers, single-cell datasets, average peaks per cell, single-cell promoters and enhancer–gene interactions for three species

	*Homo sapiens*	*Mus musculus*	*Drosophila melanogaster*	Total
Tissue/cell types	543	185	47	775
Enhancers	1 13 73 862	26 94 616	4 59 298	1 45 27 776
Single cells	10 47 052	1 30 481	19 373	11 96 906
Average peaks per cell	6212	5371	3427	5003
Promoters	39 05 212	9 42 549	94 542	49 42 303
Enhancer-gene interactions	5 08 61 554	1 14 58 766	13 38 280	6 36 58 600

Using the Jaccard matrix, we measured the weight of any single-cell dataset ${{\rm{C}}_{\rm{i}}}$ as:

$$\begin{equation*}{\rm{\ }}{w_{{{\rm{C}}_{\rm{i}}}}} = \frac{{\mathop \sum \nolimits_{j = 1}^n {J_{{C_i}{C_j}}}}}{{\mathop \sum \nolimits_{j = 1,k = 1}^n {J_{{C_j}{C_k}}}}}\ \left( {j,k \in \left[ {1,n} \right],j \ne i,j \ne k} \right)\end{equation*}$$

By combining all single cells into one cell type, the signal score of any consensus single-cell peak $i$ could be defined as:

$$\begin{equation*}{S_{consensus}} \left( i \right) = \mathop \sum \limits_{j = 1}^n {w_{{C_j}}}{S_{{C_j}}} \left( i \right)\end{equation*}$$

where the ${S_{{C_j}}}( i )$ represents the signal score of the peak $i$ in the single-cell dataset ${C_j}$.

Furthermore, we removed the single-cell peaks overlapping with promoter or exon regions. We also used the experimentally validated silencers in SilencerDB (45) as a key filter to remove the single-cell peaks that overlapped with silencers. Finally, one consensus single-cell peak should satisfy two the requirements: (i) The signal of single-cell peak should be larger than 95% of the random signals calculated by shuffling the peaks in each single cell; (ii) Cicero connection score (≥0.1) is required to display the interaction of single-cell peak with at least one gene promoter.

To evaluate the accuracy of single-cell enhancers identified by this approach, we extracted the experimentally validated active enhancers from the VISTA database (9) as the gold standard. We compared them with bulk enhancers from the EnhancerAtlas 2.0 (6) in four human tissue/cell types. For single-cell or bulk enhancers in one cell type, the ones that overlapped with VISTA enhancers were classified as the positives while the others remained as the negatives. The sensitivity and specificity of the single-cell or bulk enhancers were computed on a base pair basis. We used the area under the receiver operating characteristic (AROC) to evaluate the performance for single-cell or bulk enhancers. The results showed that single-cell enhancers in brain, heart, eye and cranial nerve had much more overlaps with VISTA enhancers than the ones in bulk enhancers, as well as an average higher performance measured by AROC than bulk enhancers (Supplementary Figure S2). This indicated that single-cell enhancers were more accurately annotated than bulk enhancers.

### Identification of enhancer–ṇpromoter interactions in scATAC-seq data

To identify the enhancer–ṇpromoter interactions in cell types, we employed the single-cell *cis*-regulatory network tool, Cicero, which had been widely used in many scATAC-seq projects to identify all the distal elements (e.g. enhancers)–promoter connections on a genome-wide basis (3,4,29–34). Because the scATAC-seq binary data for each tissue/cell type are extremely sparse, it is difficult to make accurate estimates of the co-accessibility score of chromatin accessibility loci with no normalization of the matrix (33,36). To successfully use Cicero, we transformed the binary matrix into a term frequency-inverse document frequency (TF-IDF) matrix using the latent semantic indexing (LSI) method for aggregating similar single cells to obtain denser counts in each peak (1,4). For each cell type, the binary count matrix $M$ was converted into TF-IDF matrix as following:

$$\begin{equation*}{\rm{ }}{M_{TF}} = t\left( {t\left( M \right)/colSums\left( M \right)} \right)\end{equation*}$$

$$\begin{equation*}IDF = {\rm{ }}log(1 + ncol\left( M \right) / rowSums\left( M \right)\end{equation*}$$

$$\begin{equation*}{\rm{ }}{M_{TF - IDF}} = IDF \times {M_{TF}}\end{equation*}$$

where $colSums( M )$ and $rowSums( M )$ represent the sum of each column or row of the matrix $M$,respectively, while $col( M )$ is the number of columns in $M$.

We then performed the Singular Value Decomposition (SVD) on the transformed TF-IDF matrix to reduce the dimensionality (1,4). Since the first dimension was highly correlated with the read depth, only the 2nd to 50th dimensions were passed to UMAP for 2D visualisation. Finally, Cicero used the UMAP coordinates and normalization matrix as the standard input to calculate the co-accessibility scores among peaks within a limited distance in DNA by the Graphical LASSO algorithm. Applying Cicero to each cell type across the three species with a cut-off of value >0.1, we annotated 63 658 600 enhancer–ṇpromoter connections involving 4 942 303 promoters, and 14 527 776 enhancers across 775 tissue/cell types in human, mouse and fly.

### Implementation of scEnhancer

We developed a powerful web server, scEnhancer, for single-cell analysis of enhancers and enhancer–ṇpromoter interactions. scEnhancer adopted the Linux CentOS7 with a new web configuration of nginx (1.20.1)-php (5.4.16)-mySQL (5.7.34) in a new web configuration to build the website. In addition, we employed perl (5.16.3) for the fast processing of text files with large data. Moreover, the HTML5 Canvas API and Javascript with a drawing module were utilized together to establish a genome browser for displaying enhancer/gene distributions for single-cell or consensus datasets of tissue/cell types. We also set up a two-handle slider in Canvas to scale the genomic regions. Especially, using several packages including Signac (1.2.1), Seurat (4.0.0), and ggplot2 (3.3.5) in R language (4.0.3), we successfully designed a powerful module with a function of online plotting capabilities to graphically display the differences among any selected group of tissue/cell types by single-cell clustering analysis. Several useful analytic tools on the homepage were available for users to compare single-cell enhancers at different levels. The current version of scEnhancer can run in Windows, Mac and Linux systems and supports common web browsers such as Google, Safari, Microsoft Edge and Firefox.

## RESULTS

### Database statistics

We employed a modified unsupervised learning approach and the Cicero algorithm to build the single-cell enhancer resource scEnhancer (Supplementary Figure S3). To date, scEnhancer has catalogued 775 tissue/cell types, including 14 527 776 consensus single-cell enhancers and 63 658 600 enhancer-gene interactions from 1 196 906 single cells in three species (Table 1). We overlaid SNPs from GWAS (46) or TF binding motifs from JASPAR (47) with enhancer regions and found that the single-cell enhancers were much enriched for SNPs and TF binding sites. We also summarized the number of single cells, enhancers, and enhancer-gene connections in all tissue/cell types for each species (Supplementary Tables S2–S4). The integrated cell types covered many cancers and nearly all tissues in human and mouse (Supplementary Tables S2 and S3). Most cell types display high quality with an average of >3000 peaks per cell (Supplementary Tables S2–S4). The final consensus single-cell enhancer was determined by the possible functional evidence from enhancer–ṇpromoter interactions as defined by Cicero (33). As more and more cell type-specific marker genes were identified (48), we will use these marker genes to confirm the cluster's cell type in the scATAC-seq clustering analysis to confirm the cell types of clusters and then predict single-cell enhancers even when single-cell datasets are not labeled with cell type information.

### Simple search

**Figure 1.**
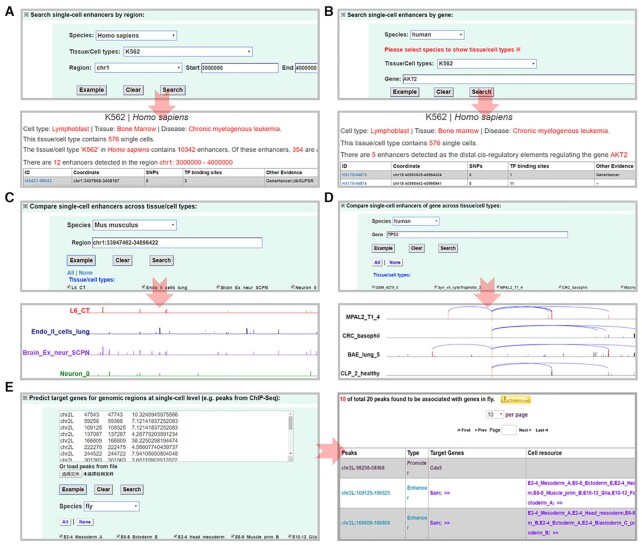
Simple search options. (**A**) Searching for single-cell enhancers by the input of a genomic region. (**B**) Querying for the enhancers around the input target gene. (**C**) Searching and comparing the distribution of single-cell enhancers across the selected tissue/cell types. (**D**) Fixing the target gene and comparing the enhancers regulating the gene across the selected tissue/cell types. (**E**) Checking whether the input genomic regions were single-cell enhancers, promoters or not in known tissue/cell types.

We designed five user-friendly analytical modules in scEnhancer for a simple search of single-cell enhancers (Figure 1): (i) Search for single-cell enhancers by region (Figure 1A). (ii) Search for single-cell enhancers by a gene (Figure 1B). (iii) Compare single-cell enhancers from different tissue/cell types (Figure 1C). (iv) Compare enhancers of a gene in different tissue/cell types (Figure 1D). (v) Predict target genes in genomic regions at the single-cell level (e.g. peaks from ChIP-Seq) (Figure 1E). Users can search for the enhancers by region in any tissue/cell of any species. In each module, an ‘Example’ button can facilitate users to give input in one-step for a simple search. We allowed the gene name or ID input from several common gene/protein resources, including Ensembl, EMBL,UCSC, PDB, FlyBase, RefSeq and UniProt (49–55). These modules serve as easy-to-use web interfaces for users to search, visualize and download single-cell enhancers and enhancer–promoter connections in any genomic region or any tissue/cell type(s).

### Advanced search

**Figure 2.**
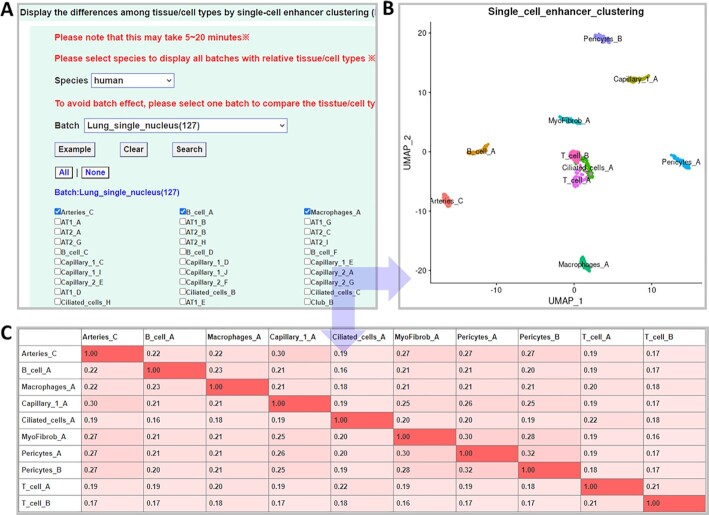
Advanced search with scATAC-seq enhancer matrix. (**A**) Browsing all tissue/cell types by clicking on the name or image of that species. (**B**) Displaying the differences among selected tissue/cell types by scATAC-seq clustering analysis. (**C**) Showing the similarities among selected tissue/cell types by the Jaccard index.

**Figure 3.**
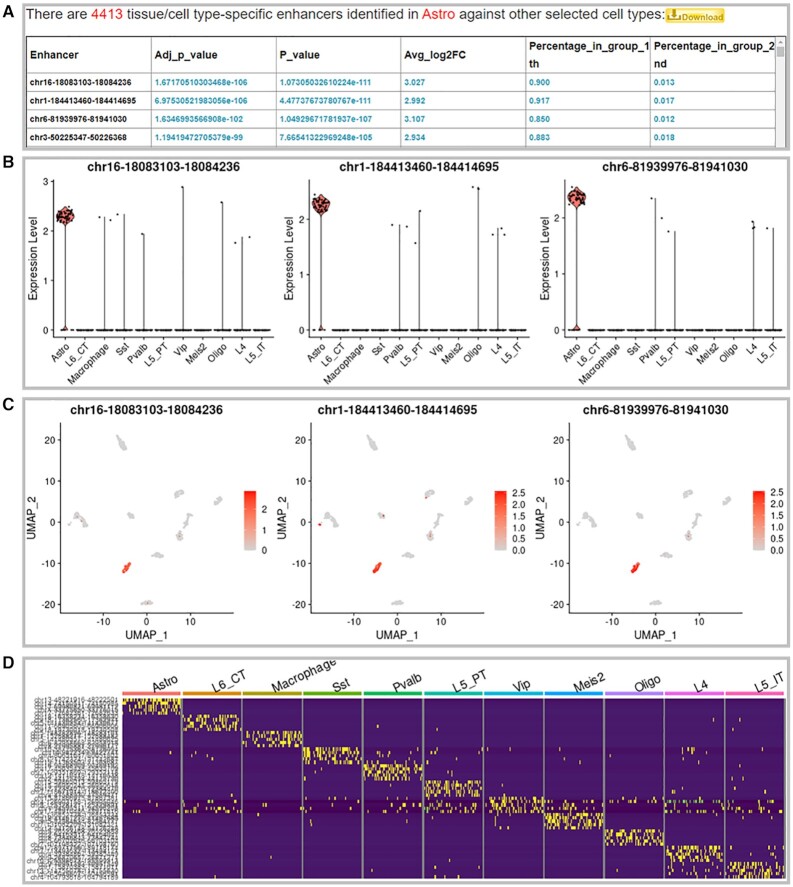
Identification of cell type-specific single-cell enhancers. (**A**) A list of cell type-specific enhancers for one cell type against the reference cell types. (**B**) Cell type specificities of the top three enhancers using VlnPlot. (**C**) The feature enrichments of the top three enhancers across all cell types using FeaturePlot. (**D**) Heatmap based on the top five single-cell enhancers that distinguish each cell type from the others.

We developed two powerful modules with several R packages as ‘advanced search’ to graphically display the differences/similarities among cell types or reveal the cell type specificity of enhancers at the single-cell level: (i) Display the differences among tissue/cell types by single-cell enhancer clustering (Figure 2). (ii) Identify tissue/cell type-specific enhancers at the single-cell level (Figure 3). To avoid the batch effects to the greatest extent, we assigned each tissue/cell type to a batch and compared different tissue/cell types within the same batch. Based on the equipment or technology platforms, we classified all the cell types into 10, 8 and 1 batches in human, mouse and fly, respectively.

In the first module, the users can select a group of cell types of interest in the same batch to observe their differences or similarities among them (Figure 2A). Merging the scATAC-seq matrices of the selected cell types, the differences among selected tissue/cell types can be displayed by DimPlot of Signac (38) (Figure 2B). In addition, this module can also calculate and present the similarities among selected tissue/cell types by Jaccard index (Figure 2C).

To reveal the cell type specificity of single-cell enhancers, the users can select a batch of interest and a primary cell type in which specific enhancers were found and select the reference cell types to compare with (Figure 3). By clicking on ‘Search’, a list of cell type-specific enhancers will be obtained (Figure 3A). Moreover, the cell-type specificity of the identified single-cell enhancers can be displayed by particular analyses, such as VlnPlot, FeaturePlot, and Heatmap (Figure 3B–D).

### Browser of single-cell enhancers

**Figure 4.**
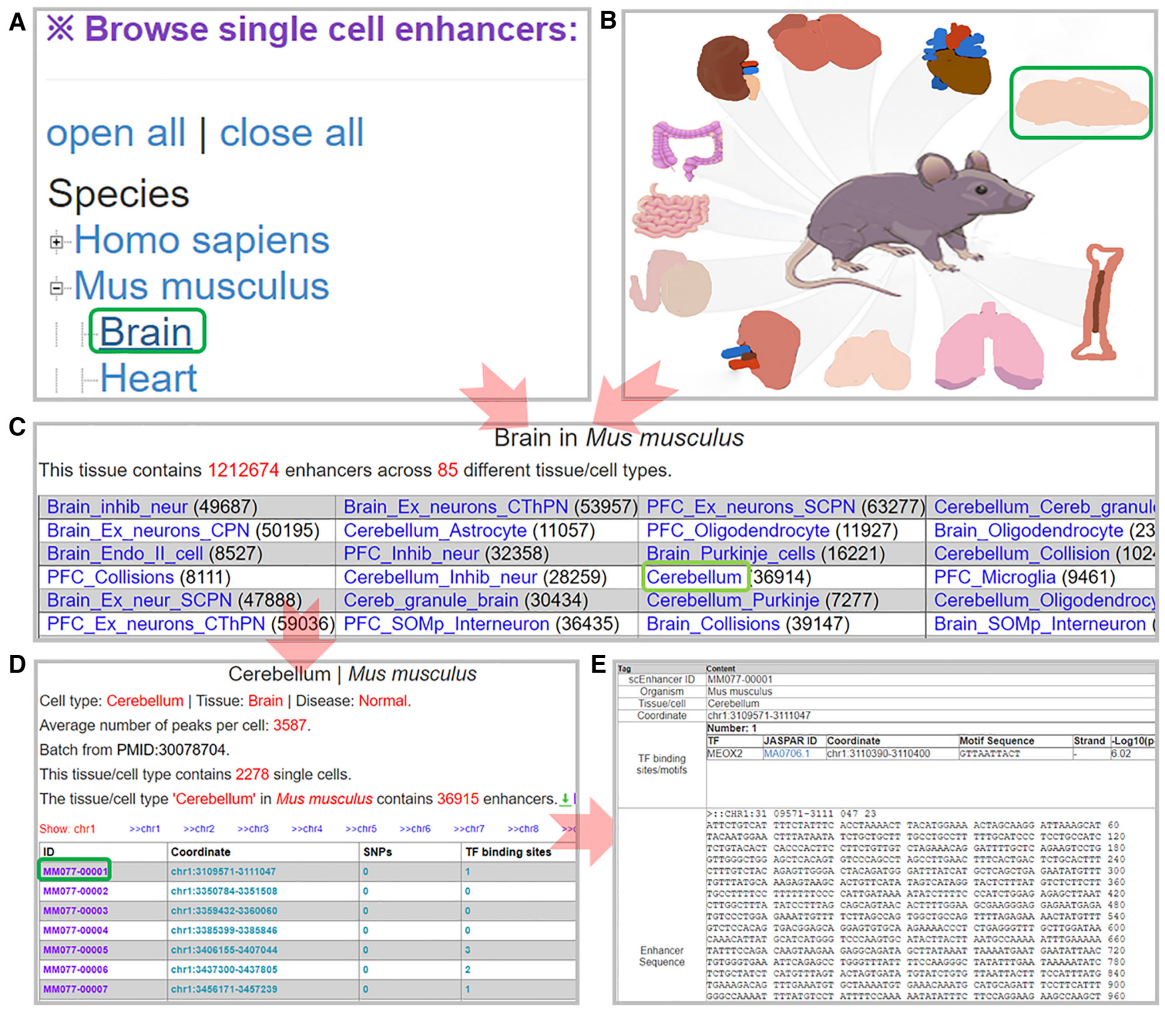
Single-cell enhancer browser. (**A**) List tissue/cell types by clicking on the name of the species or by clicking on the image of the species (**B**). (**C**) Selecting a tissue/cell type to browse all enhancers. The number in parentheses indicates the number of enhancers in that tissue/cell type. (**D**) A table of all the enhancers in the selected tissue/cell type. (**E**) A summary table describing the available features of the selected enhancer.

A browser page was provided in scEnhancer for accessing the single-cell enhancers. By clicking on the species name or image, the users can browse any tissue/cell type, any chromosome, and any single-cell enhancer, generating a summary table including the genomic coordinate of the enhancer, the contained GWAS SNPs (46), TF binding sites (47), relative super-enhancers (8), diseases (17) and DNA sequences (Figure 4).

## CONCLUSIONS

scEnhancer is the first database to annotate enhancers or enhancer–ṇpromoter interactions at the single-cell level. It contains 50 861 554, 11 458 766 and 1 338 280 enhancer–ṇpromoter connections involving 3 905 212, 942 549 and 94 542 promoters, 11 373 862, 2 694 616 and 459 298 enhancers across 543, 185 and 47 tissue/cell types in human, mouse and fly, respectively. We believe this is the most comprehensive enhancer database that includes the largest number of enhancer-related datasets at the single-cell level.

## DATA AVAILABILITY

A webserver with multiple analytic tools and deep browser capabilities is available at http://www.enhanceratlas.net/scenhancer and no login is required for all users to access the website. Tutorials for performing the scEnhancer analytic tools are freely provided at http://www.enhanceratlas.net/scenhancer/help.php. All the data including single-cell enhancers, promoters, enhancer–ṇpromoter interactions, SNPs/Motifs in enhancers, and scATAC matrix in tissue/cell types could be downloaded in http://www.enhanceratlas.net/scenhancer/download.php

## Supplementary Material

gkab1032_Supplemental_Files
