# Supplementary material for: scEnhancer: a single-cell enhancer resource with annotation across hundreds of tissue/cell types in three species
Source: Nucleic Acids Res. 2021 Nov 11;50(D1):D371–9. doi: 10.1093/nar/gkab1032 (PMC8728125; doi:10.1093/nar/gkab1032)
Supplement: gkab1032_Supplemental_Files [file gkab1032_supplemental_files.zip › scEnhancer_supplementary_figure.docx]

**Supplementary Data:**

**scEnhancer: a single-cell enhancer resource with annotation across hundreds of tissue/cell types in three species**

Tianshun Gao^1,2*^, Zilong Zheng^1^, Yihang Pan^1,2^, Chengming Zhu^2^, Fuxin Wei^3^, Jinqiu Yuan^1,2^, Rui Sun^1,2^, Shuo Fang^1,4^, Nan Wang^2^, Yang Zhou^1^, Jiang Qian^5,6^

^1^ Big Data Center, The Seventh Affiliated Hospital of Sun Yat-sen University, Shenzhen 518107, P. R. China

^2^ Scientific Research Center, The Seventh Affiliated Hospital of Sun Yat-sen University, Shenzhen 518107, P. R. China

^3^ Department of Orthopaedics, The Seventh Affiliated Hospital of Sun Yat-sen University, Shenzhen 518107, P. R. China

^4^ Department of Oncology, The Seventh Affiliated Hospital of Sun Yat-sen University, Shenzhen 518107, P. R. China

^5^ The Wilmer Eye Institute, Johns Hopkins School of Medicine, Baltimore, MD 21231, USA.

^6^ The Sidney Kimmel Comprehensive Cancer Center, Johns Hopkins School of Medicine, Baltimore, MD 21205, USA.


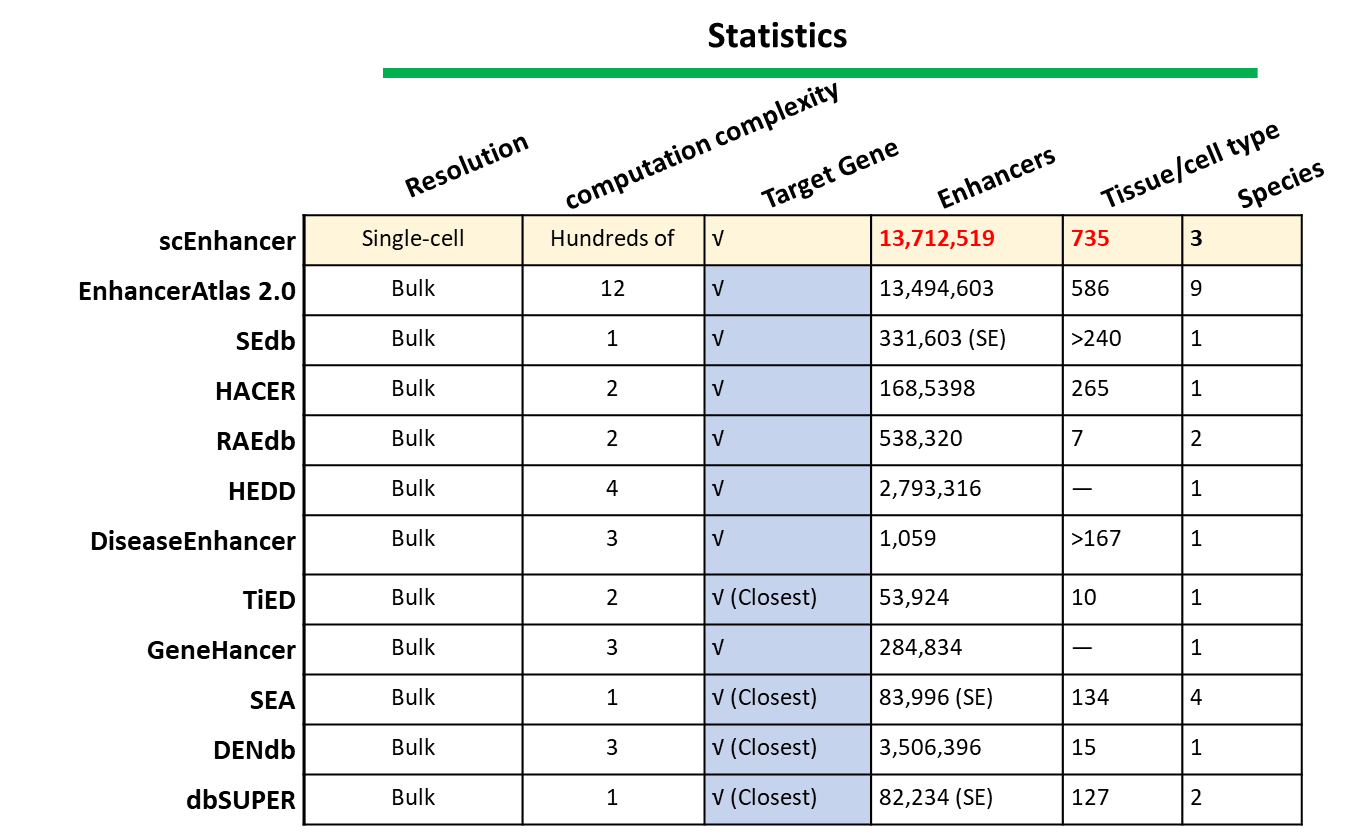


**Figure S1.** Comparison among scEnhancer and other databases. Note that the “computation complexity” here was measured by the number of datasets used in one calculation. We used the “SE” in “Statistics” for SEdb, SEA, and dbSUPER to represent the “super-enhancer”. Some databases selected the “closest” gene as the target gene, while scEnhancer used the complex algorithm Cicero to identify the target gene.

**
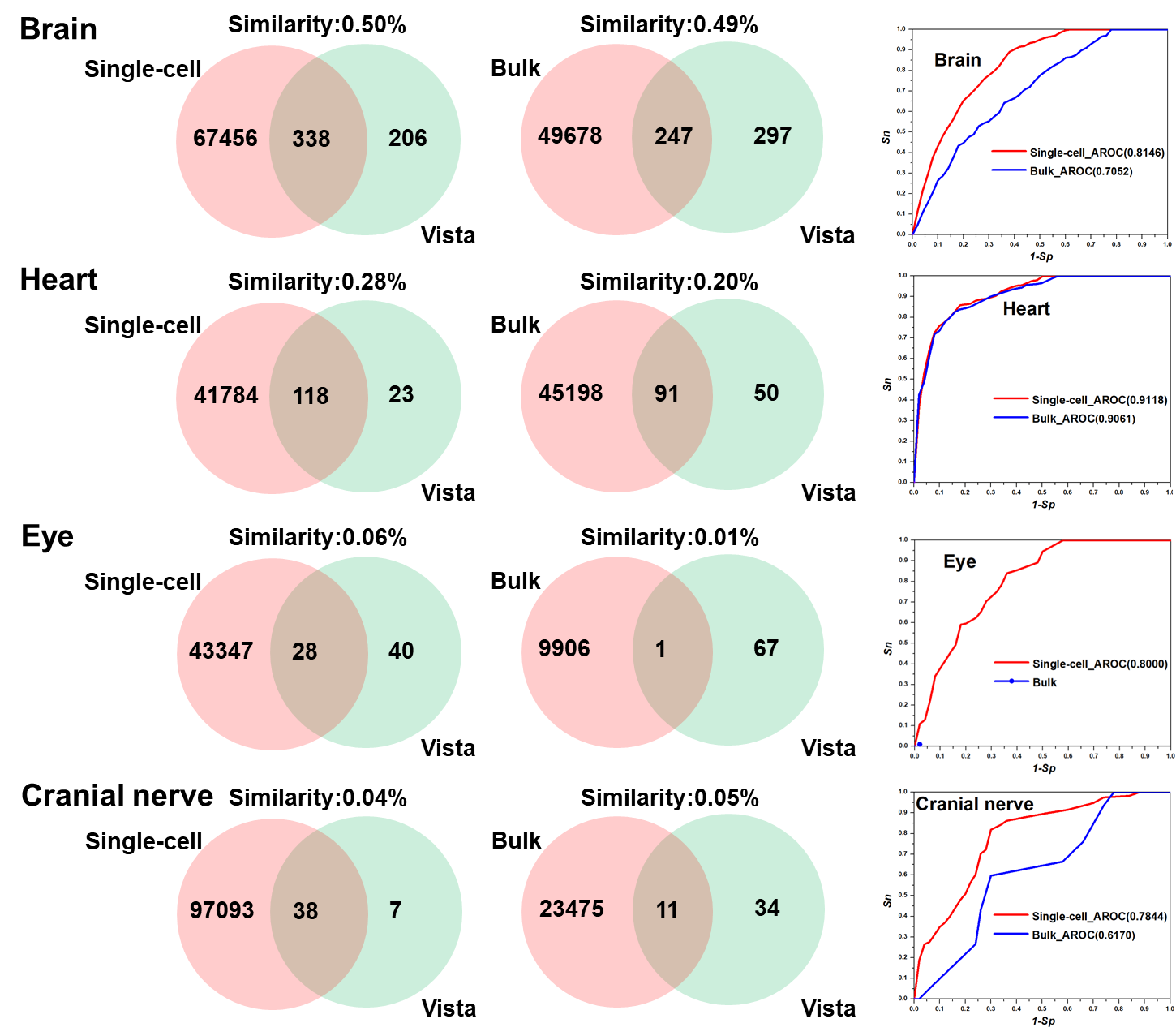
**

**Figure S2.** Comparison between single-cell and bulk enhancers. The experimentally validated enhancers from VISTA database were taken as the gold standard. Here the similarity between VISTA enhancers and single-cell or bulk enhancers was measured by the Jaccard coefficient. the sensitivity (Sn) represented the percentage of predicted active enhancers over all VISTA enhancers while the specificity(Sp) was the percentage of negative regions in the prediction space (i.e. not in VISTA enhancers), which are correctly predicted as negatives.

**
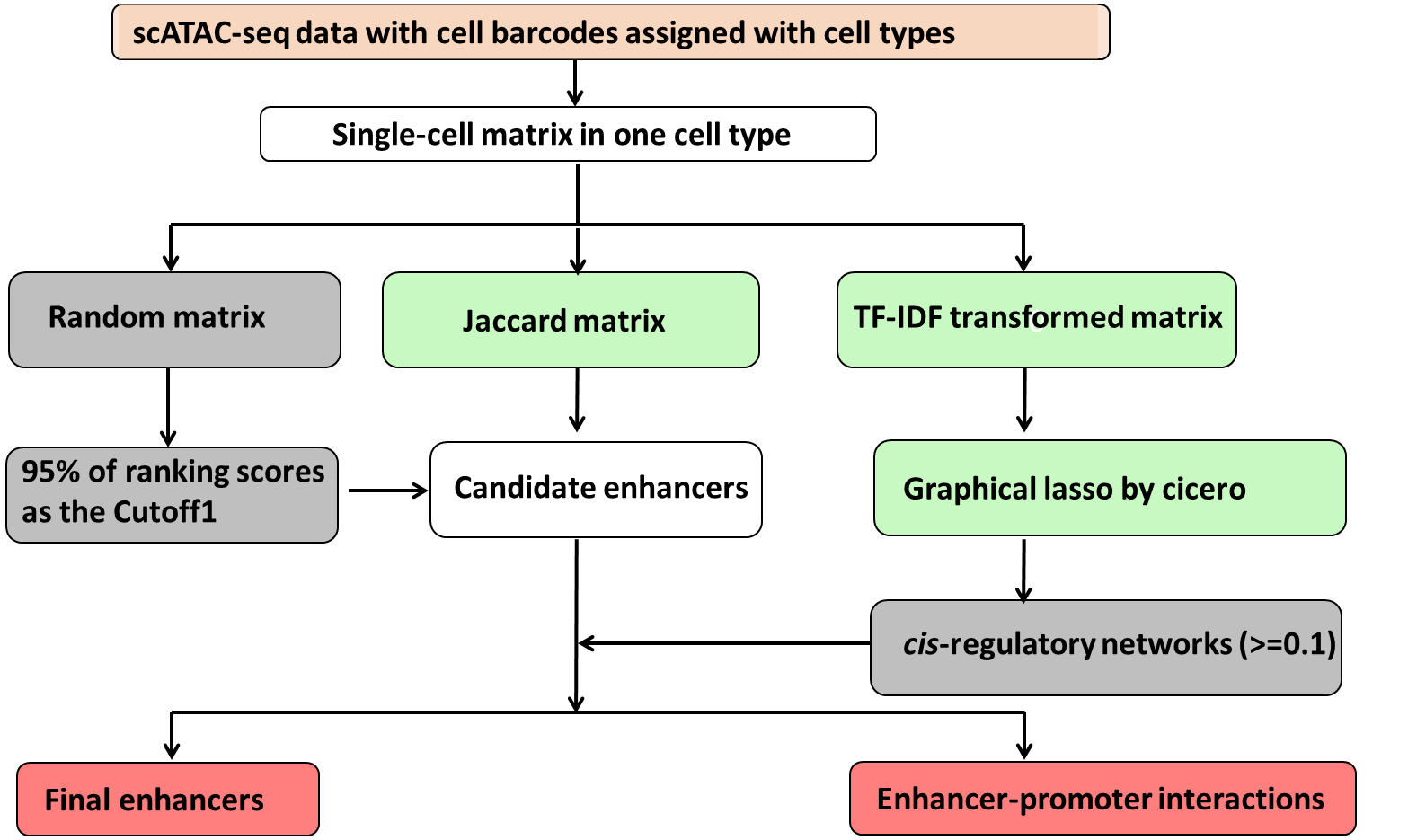
**

**Figure S3** The Overview of the scEnhancer pipeline.
