# Supplementary material for: scEnhancer: a single-cell enhancer resource with annotation across hundreds of tissue/cell types in three species
Source: Nucleic Acids Res. 2021 Nov 11;50(D1):D371–9. doi: 10.1093/nar/gkab1032 (PMC8728125; doi:10.1093/nar/gkab1032)
Supplement: gkab1032_Supplemental_Files [file gkab1032_supplemental_files.zip › scEnhancer_supplementary_table.docx]

**Supplementary Tables:**

**scEnhancer: a single-cell enhancer resource with annotation across hundreds of tissue/cell types in three species**

Tianshun Gao^1,2*^, Zilong Zheng^1^, Yihang Pan^1,2^, Chengming Zhu^2^, Fuxin Wei^3^, Jinqiu Yuan^1,2^, Rui Sun^1,2^, Shuo Fang^1,4^, Nan Wang^2^, Yang Zhou^1^, Jiang Qian^5,6^

^1^ Big Data Center, The Seventh Affiliated Hospital of Sun Yat-sen University, Shenzhen 518107, P. R. China

^2^ Scientific Research Center, The Seventh Affiliated Hospital of Sun Yat-sen University, Shenzhen 518107, P. R. China

^3^ Department of Orthopaedics, The Seventh Affiliated Hospital of Sun Yat-sen University, Shenzhen 518107, P. R. China

^4^ Department of Oncology, The Seventh Affiliated Hospital of Sun Yat-sen University, Shenzhen 518107, P. R. China

^5^ The Wilmer Eye Institute, Johns Hopkins School of Medicine, Baltimore, MD 21231, USA.

^6^ The Sidney Kimmel Comprehensive Cancer Center, Johns Hopkins School of Medicine, Baltimore, MD 21205, USA.

**Supplementary Table S1.** The description.of different extraction smethods for different data formats in scATAC-seq, including “MatrixMarket”, “H5”, “RangedSummarizedExperiment”, “Seurat RDS”, and “Raw fastq”.

**Supplementary Table S2.** The detailed information of all cell types, including the cell type description, tissue type, health state, batch source, and the numbers of single cells, average peaks per cell, enhancers, promoters, and enhancer-promoter interactions in human.

**Supplementary Table S3.** The detailed information of all mouse cell types, including the cell type description, tissue type, health state, batch source, and the numbers of single cells, average peaks per cell, enhancers, promoters, and enhancer-promoter connections.

**Supplementary Table S4.** All fly cell types with the detailed information containing the cell type description, tissue type, health state, batch source, and the numbers of single cells, average peaks per cell, enhancers, promoters, and enhancer-promoter interactions.
